# Supplementary material for: Determinants of incomplete child vaccination among mothers of children aged 12–23 months in Worebabo district, Ethiopia: Unmatched case-control study
Source: PLOS Glob Public Health. 2023 Aug 16;3(8):e0002088. doi: 10.1371/journal.pgph.0002088 (PMC10431650; doi:10.1371/journal.pgph.0002088)
Supplement: Attachment — Submitted filename: RESPONSE_TO_REVIEWERS.doc [file pgph.0002088.s002.doc]

**Rebuttal Letter**

# Manuscript Number: PGPH-D-23-00136

**Manuscript title:** Determinants of Incomplete Child Vaccination among Mothers of Children Aged 12–23 Months in Worebabo District of Amhara Region, North East Ethiopia: Unmatched Case-Control Study

**To:** PLOS ONE

**Response to editor and reviewer(s)**

Thank you for your reply regarding our manuscript and for allowing me to revised and resubmit a revised draft of our previously submitted manuscript with manuscript number PGPH-D-23-00136.

We appreciate the editor and the reviewer(s) for your precious time on the reviewing our paper and providing all acceptable comments. They were valuable and insightful comments that made improvements in the current version possible.

In the revised manuscript, we have carefully revised, modified, and considered all the comments provided by the editor and reviewer(s) as journal requirements and reviewer's comments. We tried our best to address every one of them for revision. We hope the manuscript after careful revisions meet your high standards. The authors welcome further constructive comments if any.

Below this page, we have provided the point-by-point revision and responses for all comments coded in **bold font** for each response and attempted to explain changes made in reaction succinctly. It has been improved according to the suggestion of the reviewer(s).

**Sincerely,**

Mesfin Yimer (MPH in Field Epidemiologist)
Mobile: +251912158379
Email: mesfinyimer13@yahoo.com /mesmafi13@gmail.com
Addis Abeba, Ethiopia

I. **Journal Requirements:**

Thank you for giving an opportunity to us to revise and submit the revised manuscript. The editors gave us the comments. So that, all of them have been accepted and incorporated into the revised version of this manuscript. And some of the statements also corrected in our online form.

1. Reference list revision

- We have revised the references list and we removed the references that from the retracted paper based on the journal requirement. The **removed** References from the revised manuscript are
- Galazka A, Lauer B, Henderson RH, Keja J. Indications and contraindications for vaccines used in the Expanded Programme on Immunization. Bulletin of the World Health Organization. 1984;62(3):357.
- Kim-Farley R. Global immunization. Annual review of public health. 1992;13(1):223-37.
- WHO. World Health Organization Fact sheet on immunization 2019.
- WHO. World Health Organization of Regional Office for Africa 1 in five children in Africa do not have access to life-saving vaccines.
- WHO/UNICEF. WHO/UNICEF Facts on immunization
- Jamil K, Bhuiya A, Streatfield K, Chakrabarty N. The immunization program in Bangladesh: impressive gains in coverage, but gaps remain. Health policy and planning. 1999;14(1):49-58.
- The **added** references from the revised manuscript are:
- Brenzel L, Wolfson LJ, Fox-Rushby J, Miller M, Halsey NA. Vaccine-preventable diseases. Disease control priorities in developing countries. 2006;2:389-412.
- Metcalf CJE, Andreasen V, Bjørnstad ON, Eames K, Edmunds WJ, Funk S, et al. Seven challenges in modeling vaccine preventable diseases. Epidemics. 2015;10:11-5.
- MRIA KM, Ahlstrom D. Vaccines: a life-saving choice.
- Skehan J, Muller LS. Vaccinations: Eliminating preventable illness. Professional Case Management. 2014;19(6):289-95.
- Tulchinsky TH, Varavikova EA. Communicable diseases. The New Public Health. 2014:149.
- Melaku MS, Nigatu AM, Mewosha WZ. Spatial distribution of incomplete immunization among under-five children in Ethiopia: evidence from 2005, 2011, and 2016 Ethiopian demographic and health survey data. BMC Public Health. 2020;20:1-22.
- 1Ortiz JR, Perut M, Dumolard L, Wijesinghe PR, Jorgensen P, Ropero AM, et al. A global review of national influenza immunization policies: Analysis of the 2014 WHO/UNICEF Joint Reporting Form on immunization. Vaccine. 2016;34(45):5400-5.
- Pagliusi S, Che Y, Dong S. The art of partnerships for vaccines. Vaccine. 2019;37(40):5909-19.
- Cutts FT, Izurieta HS, Rhoda DA. Measuring coverage in MNCH: design, implementation, and interpretation challenges associated with tracking vaccination coverage using household surveys. PLoS medicine. 2013;10(5):e1001404.
- Organization WH. Global vaccine action plan 2011-2020. 2013.
- Organization WH. Managing epidemics: key facts about major deadly diseases: World Health Organization; 2018.
- Mohamud AN, Feleke A, Worku W, Kifle M, Sharma HR. Immunization coverage of 12–23 months old children and associated factors in Jigjiga District, Somali National Regional State, Ethiopia. BMC Public Health. 2014;14(1):1-9.

2. Competing Interest Statement

- Many thanks for your comment and based on your comment and journal requirement, we restated the Competing Interest Statement as **“The authors have declared that no competing interest exist for this work.”**

3. Financial Disclosure Statement on Online

- Financial disclosure statement restated as: **We did not receive any funding for this study.**

4. Figure files format

- Based on the guideline of the PLOS, **we have changed the figure format to .tiff.**

5. Map Revision

- “a. Direct Link the base layer” and “b. A link the terms of uses/license information the base layer or Shape file”
- **We preferred removing the map which is labeled as “Figure 1” from the revised manuscript. Because, for this map preparation, we used the Ethiopian shape file of 2003 that our lecturer had provided for us to use for demonstration and exercise purposes during the software course. Actually, we have this shape file now but couldn’t get and link the terms of use/license information for this base layer. Due to that, it is better to remove the map from our revised manuscript. Thus, we removed the Figure 1 from our revised manuscript and there is a rearrangement of figure numbers. Consequently, “Figure 2” became “Figure 1” under sample size determination and sampling procedure technique.**

5. PLOS one journal requires all data underlying the findings described in their manuscript to be freely available to other researchers, either 1. In a public repository, 2. Within the manuscript itself, or 3. Uploaded as supplementary information. Based on the requirement

- **We have uploaded all relevant data as supplementary information.**

II. **Reviewers Comment:**

**Reviewer # 1**

The authors sincerely appreciate reviewer # 1 and would like to thank you for your all valuable, specific and helpful comments to amend and revise our manuscript. As you commented, we have revised it accordingly. So that the comments of reviewer # 1 and my revision parts are as follows:

1. **The Reviewer Comment**: The title is very long. Please revise

- My title was “Determinants of Incomplete Child Vaccination among Mothers of Children Aged 12–23 Months in Worebabo District of Amhara Region, North East Ethiopia: Unmatched Case-Control Study” and the based on the comment which is changed in to
- The authors thank this reviewer for this comment and we changed to: “**Determinants of Incomplete Child Vaccination among Mothers of Children Aged 12–23 Months in Worebabo District, Ethiopia: Unmatched Case-Control Study**”.

2. **The Reviewer’s Comment**: Reframe the sentence “as systematic identification of the factors” under introduction

- Kindly we reframed the sentence as: **Identifying the determinants of incomplete and underutilization of vaccination services is critical for developing effective strategies and overall improvement of vaccination coverage and decreasing the vaccination defaulting rate.**

3. **The Reviewer’s Comment**: Please revise the sentence “Also, the majority 224 (50.8%) of the mothers

- We thank this reviewer also and revised as: **half 224 (50.8%) of the mothers**

4. **The Reviewer’s Comment**: Please revise the sentence “The majority 200 (45.4%) of mothers/caregivers of the children in both study groups”

- The authors fully agree with the reviewer comment and we have revised this sentence accordingly as: **Nearly half 200 (45.4%) of mothers/caregivers of the children in both study group;**

5. **The Reviewer’s Comment**: “Please revise the sentence Also, the majority 224 (50.8%) of the mothers” While describing the results section,

- Thank you accepted and revised as above (repeated): **half 224 (50.8%) of the mothers**

6. **The Reviewer’s Comment**: Please discuss in the method section how you did you compute knowledge and then categorize it as good knowledge. The same for the positive attitude and positive perception.

- Thank you for this comment and accepted and revised as:
- **For knowledge, six immunization knowledge-related questions were used to assess maternal/caretaker knowledge and then correct answers received a one, while incorrect answers received a zero. After that computing the mean and those who scored >5.25 was thought to have good knowledge, while those who scored ≤5.25 was have poor knowledge.**
- **Using two Likert Scale questions, we assessed people's attitudes toward health-care institutions and their views on vaccine side effects. The mean score for each construct was calculated**
- **4.02 mean score for perception for health institution support and**
- **3.42 mean score for perception for vaccine side effect and classified as positive or negative.**

**Those who scored lower than the mean** **(≤4.02 and ≤3.42) was labeled as having negative perceptions.**

- **Three five-point Likert scale immunization-related variables were used to assess mothers' attitudes toward immunization service. Then the mean was computed and. Positive attitudes were defined as scores greater than the mean score (>10.9), while negative attitudes were scores less than the mean (≤10.9).**

7. **The Reviewer’s Comment**: Health care utilization, please specify which model that have been followed to select predictors for the outcome variables. For example, health belief model

- Thank you for your question: **We didn’t follow the theory based like health belief model for this analysis. Our analysis was guided by preparing the conceptual frame work which is prepared for this study by reviewing different literatures to identify factors or determinants for incomplete immunization. Because, we didn’t know about them (the actual determinants for incomplete immunization) in our study area. Then, also we have changed the “health care utilization” sub heading under the result part by “health service-related characteristics of mothers/caregivers”**.

8. **The Reviewer’s Comment**: Please revise the sentence “Above two-thirds of the respondents, 374 (84.8%) from both groups

- We agreed this comment and revised it as: **Almost fourth-fifths of the respondents, 374 (84.8%) from both groups (cases and controls) preferred to deliver their child at the health institute (health centers/health posts)**

9. **The Reviewer’s Comment**: Please revise the sentence “Almost all mothers 119 (81 %) of the cases and 278 (94.6%) controls who attended postnatal

- Thank you we agreed and revised as: **About 119 (81%) of mothers of the cases and 278 (94.6%) controls who attended postnatal**

10. **The Reviewer’s Comment**: Conclusion Very long. Kindly revise to make concise and shorter sentences.

- Thank you for your comment also and agreed with your comment and have revied or changed to the conclusion as:

**In conclusion, this study found that mothers or caretakers age being older (≥ 35 years), with a negative attitude towards immunization, poor knowledge, lack of antenatal care service, delivery at home, lack of visit by a health professional within a year, and spending more than an hour reaching the vaccination site are all statistically significant and independent predictors of incomplete vaccination.**

**Therefore, the district health office and health professionals should work to create awareness about maternal health services, their importance and need for complete immunization. Higher officials and health professionals should also prepare a plan and strategy to visit households at least once per month to trace the defaulters, and establish new vaccination sites to decrease long-distance travel.**

**Reviewer # 2**

The authors would like to thank reviewer # 2 for your all valuable, specific, and helpful comments to amend and revise this manuscript. Based on your comment we tried to address and revised it accordingly. So that the comments of reviewer #2 and my revision parts are as follows:

1. **The Reviewer’s Comment**: How was the selection bias avoided between the cases and controls in each Kebele? We thank you for this comment of (reviewer # 2).

- **Selection of the actual study participants (cases and controls) for this study was our critical task as participant selection procedure in order to minimize or avoid the selection bias between the cases and controls during data collection period. As a result, after selection of the Kebeles as we discussed in the method section, sampling frame (for both cases and controls) was prepared for each Kebele as main task after reviewing the routine immunization registration book of the health posts. Then, all incompletely and completely vaccinated children in the selected Kebeles were listed from the registration book as a sampling frame. Then, the study participants (both cases and controls) were then chosen or selected from each Kebele's using a systematic random sampling technique from sampling frame (not door to door of the community during data collection) to avoid the selection bias (especially control selection) during data collection.**

2. **The Reviewer’s Comment**: What was the non-response rate in the study and how did it impact your results knowing that an assumption of 10% was factored into the sample size determination.

- We appreciate the reviewer's thoughtfulness in posing this crucial query as well. **The non-response rate for this study was zero or with 100% response rate, even if we considered 10% non-response rate (27 in number) for this study during sample size calculation or determination using the exposure variable as we have discussed in the method section. So that, we revised and described about the response rate in the first paragraph of line two of our revised manuscript under the result part. The main reason for 100% response rate was that all mothers/care takers selected for this study from the sampling frame were well known by women who worked as health development army for locally available health structure and they discussed and well communicated with them (study participants) before the data collection period. Then, data collectors were provided detailed consent to the mothers/caretakes of study participants.**

3. **The Reviewer’s Comment:** Change the “Hemophiles influenza type B”

- We thank the reviewer also for this positive comment and based on the comment w**e have corrected as “Haemophilus influenza type B”**

4. **The Reviewer’s Comment**: Under study design, line 2, change ‘’amon;; to ‘’among’’

- We thank you the reviewer for asking us this important question also. **We have corrected and changed the word “amon” to “among”**

5. **The Reviewer’s Comment**: Under health care utilization, the second paragraph talks about ‘’ Above two-thirds of the respondents, 374 (84.8%) from both groups’’. Can this be rephrased since almost 85% is not two-thirds. Suggestion: ‘’Either you use the percentage directly or you use more than 4 out 5’’

- Thank you for this comment also and we have amended and stated the statement as: **Almost fourth-fifths of the respondents, 374 (84.8%) from both groups (cases and controls) preferred to deliver their child at the health institute (health centers/health posts)**

6. **The Reviewer’s Comment**: Under discussion: paragraph two, line 3 should be changed from ‘’ Thus, children who born and get care from older mothers’’ to ‘’ Thus, children born and cared for by older mothers’’ and on Line 11, change <=19 years to ≤19 years

- Thank you the reviewer and we have changed to: **Thus, children born and cared for by older mothers and <=19 years to ≤19 years**
